# 5-Azacytidine Is Insufficient For Cardiogenesis In Human Adipose-Derived Stem Cells

**DOI:** 10.1186/1477-5751-11-3

**Published:** 2012-01-06

**Authors:** Wan Kamarul Zaman Wan Safwani, Suzana Makpol, Somasundaram Sathapan, Kien Hui Chua

**Affiliations:** 1 Department of Physiology, Faculty of Medicine, Universiti Kebangsaan Malaysia Medical Centre, Kuala Lumpur, Malaysia; 2 Department of Biochemistry, Faculty of Medicine, Universiti Kebangsaan Malaysia Medical Centre, Kuala Lumpur, Malaysia; 3 Subang Jaya Medical Centre, Selangor, Malaysia

**Keywords:** 5-Azacytidine, Cardiogenic, Ischemia, Adipose, Stem Cells, Differentiation, Heart

## Abstract

**Background:**

Adipose tissue is a source of multipotent adult stem cells and it has the ability to differentiate into several types of cell lineages such as neuron cells, osteogenic cells and adipogenic cells. Several reports have shown adipose-derived stem cells (ASCs) have the ability to undergo cardiomyogenesis. Studies have shown 5-azacytidine can successfully drive stem cells such as bone marrow derived stem cells to differentiate into cardiomyogenic cells. Therefore, in this study, we investigated the effect 5-azacytidine on the cardiogenic ability of ASCs.

**Methods:**

The cardiogenic potential of ASCs was analysed by studying the morphological changes after induction, the changes in the cardiogenic genes expression i.e. GATA4, MLC-2v, MLC-2a, NKX2.5, β-MHC, α-MHC, Atrial natriuretic peptide (ANP), Connexin 43, Cardiac Troponin C, Cardiac Troponin I and myocyte enhancer factor (MEF2C) and the changes of embryonic stem cells genes expression at P5 and P10 using quantitative PCR.

**Results:**

Our results showed that the induced ASCs did not show significant morphological difference compared to the non-induced ASCs. While quantitative PCR data indicated that most cardiogenic genes and stemness genes expression level decreased after induction at P5 and P10.

**Conclusion:**

5-azacytidine is insufficient for the cardiogenic induction of the ASCs.

## 1. Background

Myocardial infarction results in the death of cardiomyocytes and these cells cannot regenerate as they are terminally differentiated. Dead cardiomyocytes are normally replaced with scar tissue. Human adipose derived stem cells (ASCs) have been shown to have the ability to differentiate into cardiomyocytes under appropriate stimuli. Furthermore, adipose tissue can be found in abundance and easily harvested, which made it an attractive alternative stem cell source especially for transplantation purposes. Transplantation of cultured cells has been proposed as a treatment for heart failure and these cells need to be cultured and expanded for a long period to achieve a sufficient number of cells. However, safety and efficacy of long-term cultured cells have become a major concern in stem cell based therapy. These issues also need to be addressed to ensure the safety of patients undergoing stem cell transplantation. The efficacy of stem cells can be evaluated by differentiation into specific cells such as cardiomyocytes. For differentiation, stem cells need to be induced in appropriate conditions and stimuli to have structural and functional differentiated cells.

Investigators have used different methods to induce the adipose derived stem cells to differentiate into cardiomyocytes. Planat-Benard et al. (2004) showed that stromal cells from adipose tissue can spontaneously differentiate into cardiomyocytes after several days indicated by contractile activity and myotube-like structures using semi solid methylcellulose containing cytokines [1]. Gaustad et al. (2004) showed that adipose derived stem cells can be differentiated into cardiomyocytes using the extracts of rat cardiomyocytes, which was indicated by the expression of cardiac specific markers [2]. However, this could be due to the transient exposure to exogenous factors that enable it to differentiate into cardiomyocytes rather than the true potential of the ASCs to differentiate [3].

5-azacytidine is a DNA methylation agent. DNA methylation is an important epigenetic mechanism, which has been reported to be involved in gene expression, chromatic modification, × chromosome inactivation, genomic imprinting and endogenic gene silencing [4]. DNA methylation is also important in maintaining pluripotency and self-renewal of stem cells. To maintain pluripotency, genes are usually activated during hypomethylation and genes that are associated with differentiation are repressed by hypermethylation [5]. Several reports have shown that 5-azacytidine can be used to induce stem cells from bone marrow [6] and adipose tissue [7] to differentiate into cardiomyocytes. 5-azacytidine has been shown to promote myogenic differentiation of bone marrow stromal cells [8]. The ASCs pretreated with 5-azacytidine before transplantation into the myocardial scar was more efficient in preserving the cardiac function [9]. However, the use of 5-azacytidine for cardiogenic differentiation is still controversial. Several investigators have shown rather contradictory evidences regarding the use of 5-azacytidine for cardiogenic potential. Zhang et al. (2007) reported that the potential of bone marrow derived stem cells to differentiate into cardiomyocytes was passage-restricted as only P4 showed the formation of myotubes that expressed cardiac specific markers after being treated with 5-azacytidine [9]. On the other hand, Liu et al. (2003) reported that rat bone marrow stromal cells cannot be induced to differentiate into cardiomyogenic cells using 5-azacytidine unless they were immortalised cells [10].

The underlying mechanism of 5-azacytidine in promoting stem cell differentiation is still poorly understood. The effect of 5-azacytidine on the stemness gene expression levels are also important to be evaluated as they can give an insight on the extent of the cardiogenic differentiation potential of ASCs. Therefore, the aim of this study was to investigate the effect of 5-azacytidine on the cardiogenic potential and the stemness characteristic of *in vitro *ASCs at P5 and P10.

## 2. Results

### 2.1) The Morphological Changes of Induced ASCs

**Figure 1:**
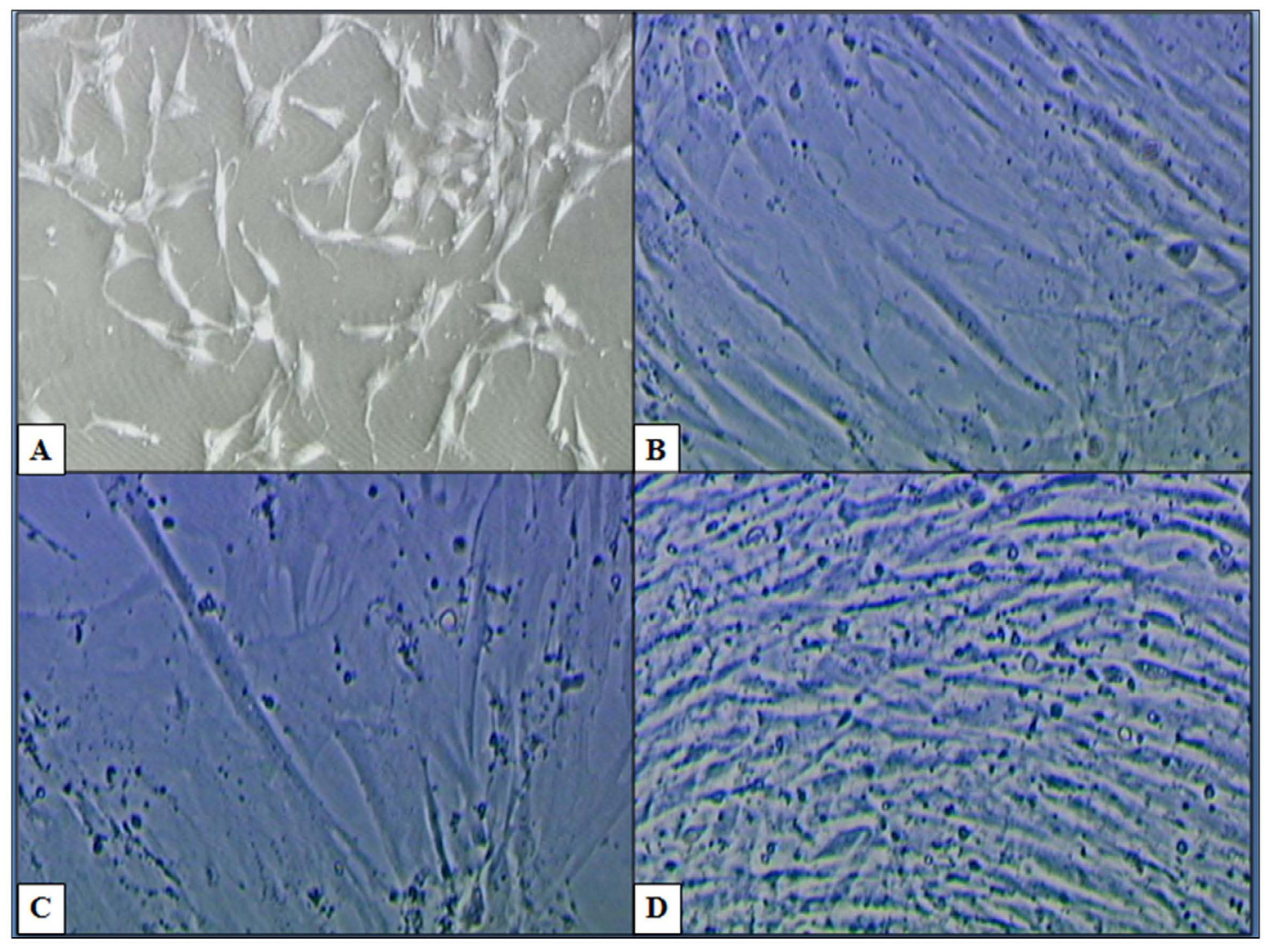
**Morphology of non-induced ASCs in basal medium which served as a control**. A) The ASCs after 24 hours of culture (50× Magnification). B) ASCs lengthened in morphology after 1 week of culture. C) and D) ASCs lengthened and increased in density after 2 and 3 weeks of culture, respectively (Magnification 200×).

**Figure 2:**
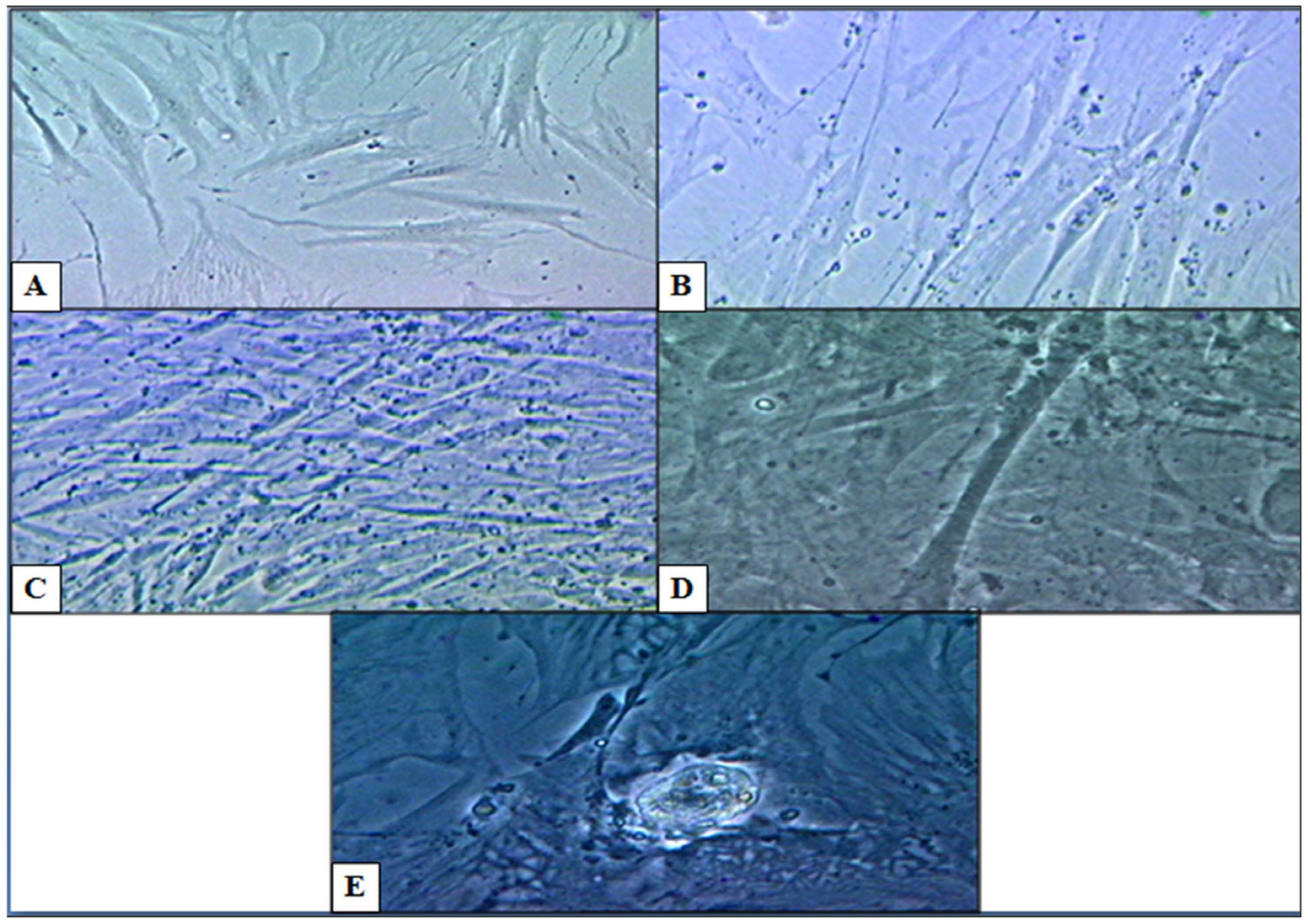
**Morphology of ASCs after undergoing cardiogenic differentiation using 5-azacytidine for 24 hours and maintained with basal medium for 3 weeks**. A) The ASCs after 24 hours of induction. B) The induced ASCs gradually increased in size after 1 week of induction. C) The induced ASCs lengthened and formed stick-like morphology at 2 weeks of induction. D) The induced ASCs formed a myotube-like structure after 2 weeks of induction. E) The ASCs formed a ball-like appearance after 2 weeks of induction but there was no spontaneous beating even after 4 weeks of induction. (Magnification 200×)

The cardiogenic induction was carried out at P5 and P10. The non-induced ASCs which were maintained in basal medium served as a control (Figure 1). Figure 1A showed the morphology of ASCs after 24 hours of culture. The ASCs lengthened in morphology after 1 week of culture (Figure 1B). While Figures 1C and 1D showed ASCs morphology after 2 weeks and 3 weeks of culture, respectively where they increased in density and lengthened to connect with the adjoining cells. After 2 days of being induced with cardiogenic medium cocktail containing 5-azacytidine, the morphology of the cells seems to change gradually (Figure 2A). The ASCs increased in size after 1 week of induction (Figure 2B). The ASCs increased in density and lengthened to form stick-like morphology at 2 weeks of induction (Figure 2C). They then connected with adjoining cells after 2 weeks to form myotube-like structures (Figure 2D). The cell population increased in density and formed a ball-like appearance after 2 weeks of induction (Figure 2E) but there was no spontaneous beating of the structure even after 4 weeks of induction as reported by Rangappa et al. (2003). However, the features of induced ASCs were similar to non-induced ASCs after 3 weeks of induction.

### 2.2) The Cardiac Specific Gene Expression Levels of ASCs

**Figure 3:**
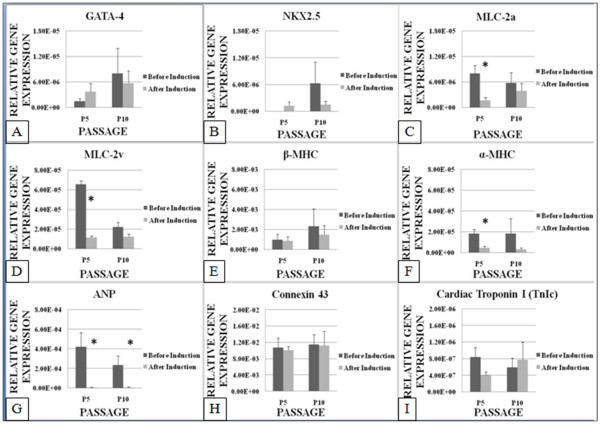
**The expression level of GATA-4, Nkx 2.5, MLC-2a, MLC-2v, β-MHC, α-MHC, ANP, Connexin 43 and TnIc of ASCs at P5 and P10 before and after induction using cardiogenic medium containing 5-azacytidine. MLC 2a, MLC-2v and α-MHC decreased significantly at P5 after induction**. ANP decreased significantly at both P5 and P10 after induction. * P < 0.05 relative to before induction by paired t-Test.

Contrary to prior findings by other investigators, quantitative PCR analysis showed that most of the cardiac specific genes of ASCs decreased in expression after being induced in cardiogenic medium for 3 weeks. GATA-4 (Figure 3A) showed a modest increase by 2 fold in expression after induction compared to before induction at P5. However, at P10, the Gata-4 expression showed no significant changes after induction compared to before induction. Nkx 2.5 (Figure 3B) showed similar pattern of expression as Gata-4 after induction. At P5, it showed a modest increase in expression compared to before induction, which was not expressed at all. At P10, Nkx2.5 showed no significant changes in its expression after induction compared to before induction.

MLC-2a (Figure 3C) expression level decreased significantly (P < 0.05) by 4 fold after induction at P5. However, no significant changes were recorded after induction at P10. MLC-2v (Figure 3D) have similar pattern as MLC-2a where it decreased significantly (P < 0.05) by 5 fold at P5 but at P10, there was no significant changes after induction. β-MHC (Figure 3E) showed no significant changes in its expression level at P5 and P10 after induction. The α-MHC (Figure 3F) expression decreased significantly (P < 0.05) in expression by 4 fold at P5 after induction. At P10, no statistically significant difference was recorded. ANP (Figure 3G) expression decreased significantly (P < 0.05) after induction at P5 and P10 by 80 fold and 40 fold, respectively. While Connexin 43 showed no significant difference in its expression at both P5 and P10 (Figure 3H).

**Figure 4:**
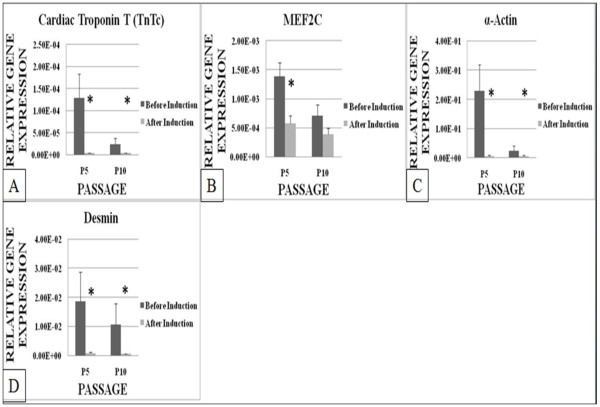
**The expression level of TnTc, MEF2C, α-actin and desmin of ASCs at P5 and P10 before and after induction using cardiogenic medium containing 5-azacytidine**. TnTc, α-actin and desmin decreased significantly at both P5 and P10 after induction. MEF2C decreased significantly at P5 after induction. * P < 0.05 relative to before induction by paired t-Test.

Cardiac Troponin I (TnIc) (Figure 3I) showed no significant difference in its expression level after induction at both P5 and P10. While Cardiac Troponin T (TnTc) (Figure 4A) expression decreased significantly (P < 0.05) after induction at P5 and P10 by approximately 30 fold and 6 fold, respectively. MEF2C (Figure 4B) showed significant (P < 0.05) decrease in expression at P5 by 2.5 fold after induction but no significant difference was recorded for MEF2C at P10. The cardiac α-actin (Figure 4C) expression decreased significantly (P < 0.05) by 40 fold at P5 and by 2 fold at P10 after induction. Desmin (Figure 4D) also decreased significantly (P < 0.05) by approximately 20 fold at both P5 and P10.

### 2.3) The Stemness Genes Expression Levels of ASCs

**Figure 5:**
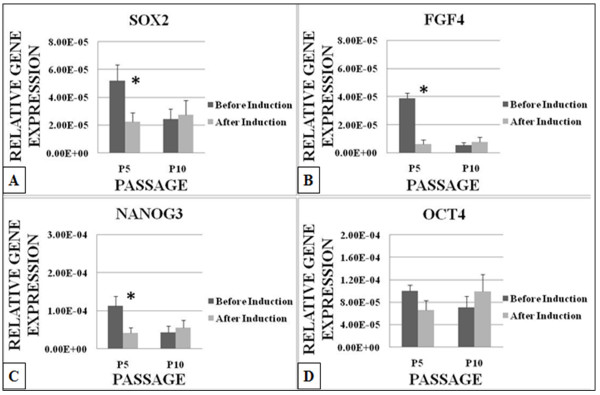
**Embryonic stem cells gene (Stemness genes) expression levels of ASCs before and after induction using cardiogenic medium containing 5-azacytidine**. Most stemness genes decreased significantly (P < 0.05) after induction at P5. * P < 0.05 relative to before induction by paired t-Test.

Quantitative PCR analysis on the stemness genes showed that Sox2 (Figure 5A) decreased significantly (P < 0.05) by 2 fold at P5 but no significant changes were recorded at P10. FGF4 (Figure 5B) decreased significantly (P < 0.05) by 6 fold after induction at P5 but no significant changes was recorded at P10. Nanog3 (Figure 5C) also decreased significantly (P < 0.05) by 3 fold at P5 after induction and no significant changes was recorded at P10. Oct4 (Figure 5D) showed no significant changes in its expression at both P5 and P10.

## 3. Discussion

The ability of 5-azacytidine to induce cardiogenic differentiation in stem cells or progenitor cells has been documented by other investigators especially using bone marrow derived stem cells. Makino et al. (1999) has successfully differentiated immortalised murine bone marrow stromal cells into cardiomyogenic cells using 5-azacytidine [11]. Xu et al. (2004) also showed that mesenchymal stem cells derived from adult human bone marrow can differentiate into cells with cardiomyocyte phenotype [6]. Rangappa et al. (2003) reported that mesencyhmal stem cells from adipose tissue of New Zealand white rabbits can be differentiated into cardiomyocytes and these transformed cells started to beat spontaneously at 3 weeks after being treated with 5-azacytidine [7]. While Burlacu et al. (2007) reported that 5-azacytidine promotes rather than induces the myogenic differentiation of bone marrow progenitor cells as it enhanced the appearance of myogenic markers [8].

In contrast, our data showed that ASCs did not differentiate into cardiomyocytes even after 4 weeks of induction. In the present study, we used the same method described by Rangappa et al. (2003) with slight modification where we induced the ASCs using the cardiogenic medium cocktail containing 5-azacytidine (10 uM) for 24 hours and the cell culture was then maintained in normal basal medium for 3 weeks. According to Rangappa et al. (2003), 24 hour incubation period using 5-azacytidine was the most effective [7]. However, the phenotype of cardiogenic cells and the conversion of ASCs into beating-cardiomyocytes as reported by Rangappa et al. (2003) were not observed in the present study. Morphologically, the only evident difference between induced and non-induced ASCS is the formation of the ball-like structure. The formation of myotube-like structure and the appearance of ball-like structure after 3 weeks of induction in the present study could be due to the post confluent state of the induced ASCs. Similar features were also observed in non-induced ASCs. In the present study, 5-azacytidine may promote cell proliferation and viability instead of differentiation into cardiomyocytes.

To analyse the molecular changes during cardiogenic induction of ASCs by 5-azacytidine, we have studied a panel of cardiac specific and non-cardiac specific gene expression levels. Data from quantitative PCR showed that most cardiogenic genes expression levels decreased after induction at P5 and P10. In cardiogenesis, GATA-4, Nkx2.5 and MEF2C were known as key regulator in cardiac development (Planat-Bernard et al, 2004). The cardiac transcription factors GATA-4 and Nkx 2.5 showed a modest increase in expression at P5 but not at P10. While MEF2C decreased at P5 and P10 after induction. Similar effect was observed for atrial myosin light chain (MLC-2a) and ventricular myosin light chain (MLC-2v), which were found in atrial and ventricular respectively were known as structural genes in cardiomyogenesis [1]. Other cardiogenic genes such as cardiac beta-myosin heavy chain (β-MHC) and cardiac alpha-myosin heavy chain (α-MHC) also known as cardiac structural genes decreased at P5 and P10. They are mostly located in the ventricles of the heart and involved in the early stage of cardiogenesis in embryonic development [12]. Connexin 43 (Cx43) is the principal connexin isoform in the mouse ventricle also decreased in expression. Cx43 provides electrical coupling between cells [13]. Atrial natriuretic peptide (ANP) is a secretion product, which is involved in the later stage of cardiac development [1] decreased at P5 and P10 after induction. Cardiac Troponin I (TnIc) and Cardiac Troponin T (TnTc) were also structural genes showed no significant changes in expression after induction compared to before induction. Cardiac α-actin and desmin [14], which was found mainly in smooth muscle also decreased after induction at P5 and P10. This result showed that the cardiogenic medium containing 5-azacytidine used in this study is not sufficient to increase the expression of cardiac specific genes in ASCs.

In the present study, we speculate that 5-azacytidine alone may not be sufficient to support the cells to differentiate into cardiomyocytes. Similar result was reported by Lee et al (2009) who demonstrated that 5-azacytidine alone cannot induce the ASCs to differentiate into cardiomyocytes especially at the early stages of cardiomyogenic [3]. A study by Martin-Rendon et al. (2008) also showed that 5-azacytidine-treated MSCs derived from umbilical cord, cord blood and bone marrow did not generate cardiomyocytes *in vitro *[15]. Although 5-azacytidine has been shown to induce cardiac-like phenotypes in MSCs derived from bone marrow by activating a number of genes, its effect may not be specific [15]. The use of growth factors and cytokines may be needed to induce cardiomyogenic differentiation as shown by Planat-Barnard et al. (2004) [1]. In this study, we have added basic fibroblast growth factor (bFGF) in the induction medium but it did not have any effect on the cardiogenic potential of ASCs. The bFGF may only support the cells proliferation and viability of ASCs *in vitro *instead of the cells differentiation mechanism. Data from Rosca and Burlacu (2011) showed that treatment with 5-azacytidine may promote subsequent cardiac differentiation but it is dependent on finding the adequate conditions for cardiomyogenic differentiation [16]. Correct concentration of 5-azacytidine coupled with the use of growth factors and cytokines may be able to create an adequate conditions for cardiomyogenic differentiation.

To evaluate the efficiency of ASCs differentiation during cardiogenic induction, we have also analysed the embryonic stem cell gene expression levels i.e. Nanog3, Sox2, Oct4 and FGF4 after cardiogenic induction. These genes showed a significant decrease at P5 but no significant changes were recorded at P10. While Oct4 showed no significant changes at both passage. These genes play an important role in maintenance and differentiation of embryonic stem cells, and the levels and activity of these transcription factors are indicators of embryonic stem cells pluripotent or differentiation status [17]. These genes also can be used to determine the stemness properties of adult stem cells such as ASCs [18]. Tay et al. (2008) reported that attenuation of Nanog3, Oct4 and Sox2 promotes the differentiation of embryonic stem cells [19]. In the present study, the decrease of expression levels of Nanog3, FGF4, and Sox2 indicates that there may be a differentiation activity happening at P5. Sox2 was reported to reduce during differentiation from mesodermal to hematopoietic progenitor cells [20]. The type of cell differentiation that may happen in the present study is still uncertain. Further investigation is needed to elucidate the differentiation mechanism in MSCs treated with 5-azacytidine because 5-azacytidine has been shown to induce osteogenesis [21], adipogenesis and chondrogenesis [16] in bone marrow derived stem cells. While at P10, 5-azacytidine did not affect the embryonic stem cells gene expression levels in the inducted ASCs.

## 4. Conclusion

In conclusion, 5-azacytidine is not effective on the cardiogenesis of ASCs. Further investigation is needed to elucidate adequate conditions to differentiate the ASCs into mature cardiogenic cells.

## 5. Materials and Methods

### 5.1) Isolation of ASCs From Adipose Tissue

Approval to conduct this research was obtained from the Research Committee of Universiti Kebangsaan Malaysia Medical Centre (UKMMC) (Approval No: 02-01-02-SF0290). The ASCs were harvested from lipoaspirate tissue, which was obtained from 6 consented patients (40-60 years of age) undergoing intraoperative suction lipectomy at the Subang Jaya Medical Centre, Malaysia. The lipoaspirate tissue was digested in 0.3% Collagenase Type I solution (Sigma-Aldrich, St Louise, MO) for 2 hours at 37°C. The isolated cells (P0; approximately 1,000 adherent cells) were then cultured in basal medium containing Dulbecco's Modified Eagle Medium (DMEM)/Ham F12 medium supplemented with 10% fetal bovine serum (FBS) (Gibco), 1% antibiotic-antimycotic (Gibco), 1% glutamax (Gibco) and 1% vitamin C (Sigma-Aldrich, St Louise, MO). The ASCs were incubated and maintained at 37°C with 5% carbon dioxide with medium changed every 3 days. The ASCs were grown from the initial plating and harvested using 0.125% trypsin-EDTA (Gibco) at P5 and P10.

### 5.2) Cardiogenic Differentiation of ASCs and Morphological Evaluation

The cardiogenic differentiation capability of cultured ASCs at P5 and P10 was tested using medium containing an equal volume Ham's F12 medium and Dulbecco's Modified Eagle Medium (F12:DMEM) (Gibco Invitrogen, USA) supplemented with 10% (v/v) fetal bovine serum (Gibco Invitrogen), 5% (v/v) human serum, 1% (v/v) antibiotic antimycotic (Gibco Invitrogen), 1% (v/v) glutamax (Gibco Invitrogen), and 1% (v/v) vitamin C (Sigma, St Louis, MO), 10 uM 5-azacytidine (Sigma), 5 uL of 10 ug/L of bFGF (Peprotec, USA) and 5 uL of 0.25 mg/ml amphotericin (Sigma).

Cells were seeded at 10000 cells/cm^2 ^in a culture flask with 25 cm^2 ^surface area (Orange Scientific, Belgium) and maintained in 5% CO_2 _incubator at 37°C under 95% humidity. After 24 hours of induction using cardiogenic medium, the cell cultures were maintained in normal basal medium (An equal volume Ham's F12 medium and Dulbecco's Modified Eagle Medium (Gibco Invitrogen) was supplemented with 10% (v/v) fetal bovine serum (Gibco Invitrogen), 5% (v/v) human serum, 1% (v/v) antibiotic antimycotic (Gibco Invitrogen), 1% (v/v) glutamax (Gibco Invitrogen), and 1% (v/v) vitamin C (Sigma) for 3 weeks. The medium was changed every 3 days. Cells were examined everyday by using inverted phase contrast microscope for cells growth and morphological changes. Photographs were taken to record the changes in cell morphology.

### 5.3) RNA Extraction

Total RNA was extracted from the non-induced and induced ASCs cultured at P5 and P10. The RNA was homogenised and extracted using TRI reagent (Molecular Research Center, Cincinati, OH). Chloroform was used for phase separation of the sample. Total RNA from the sample was precipitated by using absolute isopropanol (Sigma) and polyacryl carrier (Molecular Research Center) was added to the RNA which, cause the sedimentation of the RNA extract. The RNA extract was then centrifuged at 12000 rpm for 8 min at 4°C. 75% Ethanol was used to wash the extract and dried for 20 min. RNase and DNase free distilled water (Gibco Invitrogen) was used to solubilise the RNA extract.

### 5.4) cDNA Synthesis

The solubilised RNA extracts from ASCs cultured at P5 and P10 were used to synthesise cDNA by using SuperScript III First-Strand Synthesis SuperMix kit for two-step quantitative RT-PCR (Invitrogen). The synthesis was carried out according to the optimised protocol recommended by the manufacturer. The protocol conditions were 10 min at 23°C, 60 min at 42°C and 10 min at 94°C. The synthesised cDNA was used to perform Real time PCR to evaluate the expression level of the stemness genes.

### 5.5) Cardiogenic and Stemness Gene Expression Analysis Using Quantitative PCR

The non-induced and induced ASCs cultured at P5 and P10 were analysed for cardiogenic genes expression i.e. Gata4, MLC-2v, MLC-2a, Nkx2.5, β-MHC, α-MHC, Atrial natriuretic peptide (ANP), Connexin 43, Cardiac Troponin C (TnIc), Cardiac Troponin I (TnTc) and myocyte enhancer factor (MEF2C) (Table 1) and stemness genes expression level i.e. Sox-2, Nanog3, FGF4 and Oct-4 (Table 2). GAPDH was used as the housekeeping gene. Primers for each gene were designed by using Primer 3 software based on the published GeneBank database sequences. The PCR reaction was carried out using SYBR Green as an indicator in BioRad iCycler PCR machine. The reaction mixture consisted of iQ SYBR Supermix, forward and reverse primers (500 nM each), deionised water and 2 ul cDNA. The PCR reaction conditions were cycle 1: Step 1 95°C for 3 min, cycle 2 (40x): Step 1 95°C for 10 sec and Step 2 61°C for 30 sec, cycle 3: Step 1 95°C for 1 min, cycle 4: Step 1 55°C for 1 min and cycle 5: Step 1 60°C for 10 sec. Expression level of each targeted gene was normalised to GAPDH. The specificity of the primers and PCR protocol were confirmed with melting curve analysis and further verified by 2% agarose gel electrophoresis.

**Table 1:** Description of cardiogenic gene primers used in quantitative PCR

Gene	Accession No	Primer 5' 3'	PCR product size (bp)
**GATA-4**	NM_002052	**R **5'-CTCCTACTCCAGCCCCTACC-3'	207
		**F **5'-GTGGACATAGCCCCACAGTT-3'	
**MLC-2a**	NM_021223	**R **5'-GCAGACCTGAGGGAGACCTAC-3'	135
		**F **5'-ATTGAGCTTCTCCCCAAAGAG-3'	
**MLC-2v**	NM_000432	**R **5'-ACAGGGATGGCTTCATTGAC-3'	192
		**F **5'-ATGCGTTGAGAATGGTTTCC-3'	
**Nkx2.5**	NM_004387	**R **5'-GAGAAGACAGAGGCGGACAA-3'	187
		**F **5'-AGATCTTGACCTGCGTGGAC-3'	
**β-MHC**	NM_000257	**R **5'-TGATCTGGAGCTGACACTGG-3'	196
		**F **5'-CAGGGTGTTGACCTTGTCCT-3'	
**α-MHC**	NM_002471	**R **5'-CCACCCAAGTTCGACAAGAT-3'	127
		**F **5'-CACAGAAGAGGCCCGAGTAG-3'	
**ANP**	NM_000906	**R **5'-GCATTGAGCTGACACGAAAA-3'	219
		**F **5'-CCTTGACGATGTCATTGGTG-3'	
**Connexin 43**	NM_000165	**R **5'-GGACATGCACTTGAAGCAGA-3'	103
		**F **5'-GATGATGTAGGTTCGAAGCA-3'	
**TnIc**	NM_000363	**R **5'-CTCCAACTACCGCGCTTATG-3'	116
		**F **5'-CCAGCTCTTGCTTTGCAATC-3'	
**TnTc**	NM_000364	**R **5'-CATGGAGAAGGACCTGAATGA-3'	108
		**F **5'-CGTCTCTCGATCCTGTCTTTG-3'	
**MEF2C**	NM_002397	**R **5'-CAGTCATTGGCTACCCCAGT-3'	221
		**F **5'-GCAGATGGTGGCATGTTATG-3'	
**Desmin**	NM_001927	**R **5'-GCCTCATCAGGGAATCGTTA-3'	129
		**L **5'-AGGCAGCCAACAAGAACAAC-3'	
**α-Actin**	NM_005159	**R **5'-AGGGGCTGGAAGAGTGTCTCA-3'	136
		**L **5' -GCCCTGGATTTTGAGAATGA-3'	

**Table 2:** Description of stemness gene primers used in quantitative PCR

Gene	Accession No	Primer 5' 3'	PCR product size (bp)
**GAPDH**	NM_002046	**R **5'-GGAGGAGTGGGTGTCGCTGT-3'	217
		**F **5'-TCCCTGAGCTGAACGGGAAG-3'	
**Sox2**	NM_003106.2	**R **5'GGTAGTGCTGGGACATGTGAA-3'	132
		**F **5'-TTACCTCTTCCTCCCACTCCA-3'	
**FGF4**	NM_002007	**R **5'-GGTTCCCCTTCTTGGTCTTC-3'	118
		**F **5'-GATGAGTGCACGTTCAAGGA-3'	
**Nanog3**	NM_024865.2	**R **5'-TGTTTGCCTTTGGGACTGGT-3'	153
		**F **5'CTGTGATTTGTGGGCCTGAA-3'	
**Oct-4**	NM_002701	**R **5'-GAAGTGAGGGCTCCCATAGC-3'	180
		**F **5'-AAGGATGTGGTCCGAGTGTG-3'	

### 5.6) Statistical Analysis

Data was analysed by using SPSS 15.0 (SPSS Inc., Chicago, IL) and subjected to paired t-test to determine significance difference (P < 0.05) of the gene expression levels between passage P5 and P10. Data were presented as the mean ± standard error of mean (SEM).

## 6. Abbreviations

ANP: Atrial Natriuretic Peptide; bFGF: Basic Fibroblast Growth Factor; TnIc: Cardiac Troponin I; TnTc: Cardiac Troponin T; FGF4: Fiborblast Growth Factor 4; GAPDH: Glyceraldehyde-3-Phosphate Dehydrogenase; ASCs: Human Adipose-Derived Stem Cells; MSCs: Mesenchymal Stem Cells; α-MHC: α-Myosin Heavy Chain; β-MHC: β-Myosin Heavy Chain; MLC-2a: Atrial Myosin Light Chain-2a; MLC-2v: Ventricular Myosin Light Chain-2v; MEF2C: Myocyte Enhancer Factor; PCR: Passage (P), Polymerase Chain Reaction.

## 7. Competing interests

The authors declare that they have no competing interests.

## 8. Authors' contributions

WKZWS - Carried out the cell culture work, morphological study, analysis of β-galactosidase activity and quantitative RT-PCR study, counting of PDT, analysis of cell cycle and CD markers using flow cytometer. Performed statistical analysis. Involved in manuscript drafting and data analysis. SM - Participated in the design of the study, data analysis and manuscript drafting. SS - Participated in the design of the study, involved in getting patients' consent, carried out lipectomy procedure. KC - Participated in the design of the study, manuscript drafting, data analysis, revising the manuscript and gave final approval for publication. All authors had read and approved the final manuscript.
